# Dirichlet Multinomial Mixtures: Generative Models for Microbial Metagenomics

**DOI:** 10.1371/journal.pone.0030126

**Published:** 2012-02-03

**Authors:** Ian Holmes, Keith Harris, Christopher Quince

**Affiliations:** 1 Department of Bioengineering, University of California, Berkeley, California, United States of America; 2 School of Engineering, University of Glasgow, Glasgow, United Kingdom; Argonne National Laboratory, United States of America

## Abstract

We introduce Dirichlet multinomial mixtures (DMM) for the probabilistic modelling of microbial metagenomics data. This data can be represented as a frequency matrix giving the number of times each taxa is observed in each sample. The samples have different size, and the matrix is sparse, as communities are diverse and skewed to rare taxa. Most methods used previously to classify or cluster samples have ignored these features. We describe each community by a vector of taxa probabilities. These vectors are generated from one of a finite number of Dirichlet mixture components each with different hyperparameters. Observed samples are generated through multinomial sampling. The mixture components cluster communities into distinct ‘metacommunities’, and, hence, determine envirotypes or enterotypes, groups of communities with a similar composition. The model can also deduce the impact of a treatment and be used for classification. We wrote software for the fitting of DMM models using the ‘evidence framework’ (http://code.google.com/p/microbedmm/). This includes the Laplace approximation of the model evidence. We applied the DMM model to human gut microbe genera frequencies from Obese and Lean twins. From the model evidence four clusters fit this data best. Two clusters were dominated by Bacteroides and were homogenous; two had a more variable community composition. We could not find a significant impact of body mass on community structure. However, Obese twins were more likely to derive from the high variance clusters. We propose that obesity is not associated with a distinct microbiota but increases the chance that an individual derives from a disturbed enterotype. This is an example of the ‘Anna Karenina principle (AKP)’ applied to microbial communities: disturbed states having many more configurations than undisturbed. We verify this by showing that in a study of inflammatory bowel disease (IBD) phenotypes, ileal Crohn's disease (ICD) is associated with a more variable community.

## Introduction

Next generation sequencing, applied to microbial metagenomics, has transformed the study of microbial diversity. Microbial metagenomics, or sequencing of DNA extracted from microbial communities, provides a means to determine what organisms are present without the need for isolation and culturing, which can access less than 1% of the species in a typical environment [1]. Prior to next generation sequencing individual DNA fragments from a sample were cloned and then Sanger sequenced [2] – a procedure that is slow and expensive when done on a per read basis. Direct next generation sequencing, for example 454 pyrosequencing [3] or Illumina [4], is cheaper and faster, which has allowed much larger studies of microbial diversity, with more reads in total, and with more communities sampled. However, the development of statistics to extract ecologically meaningful information from these data sets has not developed as quickly as the experimental methodology. In particular, tools that can account for the discrete nature, sparsity, and variable size of these data sets are lacking. We propose the Dirichlet multinomial mixture as a generative modelling framework that addresses this need.

Broadly, microbial metagenomics data can be of two types: either amplicons or shotgun metagenomics. Amplicons are generated by PCR amplification of a specific marker gene region – typically a variable region from the 16S rRNA gene – prior to sequencing, so that the data consists of reads from homologous genes in different organisms. In shotgun metagenomics DNA is fragmented in some way and those fragments sequenced, generating reads from throughout the genome of the different community members. For both amplicons and shotgun reads it is possible to classify sequence reads against known taxa, and determine a list of those organisms that are present and the read frequency associated with them [5]. For the majority of environments, many organisms will not have been taxonomically classified and sequenced before, in which case the list of taxa may have to be generated at a low resolution phylogenetic level, e.g. phylum, to achieve a reasonable proportion of classified reads. Alternatively, an unsupervised strategy can be used to identify proxies to traditional taxonomic units by clustering sequences, so called Operational Taxonomic Units (OTUs) [6]. This is commonly performed in the case of homologous marker genes from amplicons but can also be applied to shotgun metagenomics data [7]. Whether supervised or unsupervised approaches are used the end result is the same: a community is represented by a list of types, either taxa or OTUs, and their frequency. For shotgun metagenomics data much more analysis is possible, utilising information about the function of genes that are sequenced, but here we will focus on the analysis of community structure generated by microbial metagenomics. Typically, this will be generated as amplicons, which typically will be 454 pyrosequenced, but we would emphasise that the approach can be applied to any list of taxa or OTUs with discrete abundances.

Early studies of microbial communities focussed on cataloguing diversity in individual samples, asking: how many different taxa or OTUs were present [8], [9]? A striking result was that the observed diversity was very high, and that most species were observed with low abundance; this phenomenon has been termed the ‘rare biosphere’ [8]. These early studies ignored the impact of sequencing and PCR errors which can inflate OTU diversities [10], but even after the application of algorithms capable of removing those errors [11], observed diversities remain high in most environments and abundances are still skewed to low abundances in almost all [10], [12]. The consequence of this is that even with very large read numbers we will have only sampled a fraction of the true diversity [13].

The natural extension to examining the diversity in an individual sample is to look at patterns across samples from similar environments. Barcoding allows multiple samples to be sequenced in a single run but difficulties quantifying DNA concentration means that the number of reads from each sample will usually vary substantially [14]. Sub-sampling can be used to reduce all samples to the same size but that inevitably throws away large amounts of meaningful data. The majority of studies have used exploratory statistics to search for natural patterns in the data, unsupervised learning again. A common strategy is to use multivariate ordination techniques, where samples are positioned in a space of reduced dimensionality so as to preserve the distances between them in the original higher dimensional space; often two or three dimensional ordinations are used and then it is possible to look for patterns by eye. A classic example of an ordination method is principal components analysis (PCA), which generates new dimensions that are linear combinations of the original, chosen so as to preserve the Euclidean distance between samples [15]. Euclidean distances are not very appropriate for microbial community analysis, much better is to use measures that incorporate the phylogentic divergence between types, e.g. Unifrac [16]. Ordination can be performed with arbitrary distance metrics using multidimensional scaling methods, these can be either metric in that they preserve distances or non-metric in that they preserve the ranking of the distances. An example of a metric multidimensional scaling is principal coordinates analysis which has proven a useful and popular tool when coupled with Unifrac for exploratory data analysis [17].

Clustering is another means of exploratory data analysis which searches for natural groups or partitions in the samples. Hierarchical clustering, where a tree of relationships is generated without explicitly grouping samples unless an arbitrary cut-off is chosen, is quite commonly used in microbial community analyses, partitional clustering where the samples are divided into groups has traditionally been less popular. This may be because of the need to decide *a priori* how many clusters are present. Generally variants of the 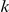-means algorithm have been used together with heuristics to decide how good a clustering is. To date there has been no model based clustering of microbial community data. This question of the natural number of types of communities has received particular attention recently in the context of the human gut, for which it has been suggested that three microbial community types, known as envirotypes (or, in the context of the gut, enterotypes) are to be found [18]. Classification, or supervised learning, is closely related to clustering, except here the problem is not to find natural groups in the data but to predict the group of a new sample, given a labelling of samples in a training data set. Two studies applying classification methods to microbial communities have appeared recently [19], [20]. Most of the algorithms used were, as for the unsupervised approaches, developed for continuous data with the notable exception of the multinomial naive Bayes (MNB) model in Knights et al. (2001) [20].

There are, however, problems inherent in using standard multivariate techniques for the analysis of microbial metagenomics data. The data, even if normalised into relative abundances, is fundamentally discrete and can only be approximately modelled by continuous variables. In addition, the high diversity (relative to sampling effort) results in very sparse data sets; most taxa appear in only a few samples at low abundance. Finally, the samples vary in read number: a small sample will inherently be more noisy than a larger one. All these issues can be addressed using an explicit sampling scheme. Instead of viewing the sample as representing the community, we view it as having being generated by sampling from the community. The most natural assumption to make is sampling with replacement, so that the likelihood of an observed sample is a multinomial distribution with a parameter vector where a given entry represents the probability that a read is from a given taxa. These probabilities in the limit of very large community sizes will become the relative frequencies of the taxa. This provides a discrete model, that accounts for different sample sizes, and can model sparse data.

We will show how this multinomial sampling can be used as a starting point for a generative modelling framework, one that explicitly describes a model for generating the observed data [21]. This provides model-based alternatives for both clustering and classification of microbial communities. The natural prior for the parameters of the multinomial distribution is the Dirichlet. This is a probability distribution over probability vectors. In the context of microbial communities we can view it as describing a metacommunity from which communities can be sampled. Its parameters then describe both the mean expected community and the variance in the communities. As we will show, one of the major advantages of the Dirichlet prior is that the community parameter vectors which are unobserved can be integrated out or marginalised to give an analytic solution to the *evidence*: the probability that the data was generated by the model. By extending the Dirichlet prior to a mixture of Dirichlets [22]–[24], so that the data set is generated not by a single metacommunity but a mixture of multiple metacommunities, we obtain both a more flexible model for our data and a means to cluster communities. To perform the clustering, we simply impute for each sample the component which is most likely to have generated it. This separates samples into groups according to the metacommunity it has the highest probability of deriving from. The advantage of this approach over simple 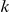-means type strategies is twofold: (1) the clusters can be of different sizes depending on the variability of the metacommunity, and more importantly (2) because we now have an explicit probabilistic model that is appropriate to the data, then we can use the evidence together with methods to penalise model complexity to provide a rigorous means of determining optimal cluster number.

Multinomial sampling has been used previously in the study of microbial communities [20], and it has been coupled with a Dirichlet prior [25], but the extension of that prior to a mixture of Dirichlet components in this context is completely novel, as is the explicit association of each Dirichlet component with a different metacommunity. The major challenge for our framework is how to fit the Dirichlet mixture given the very large dimensionality of microbial metagenomics data sets. This will make Gibbs sampling to obtain posterior distributions for the Dirichlet parameters challenging, at least for OTU based data sets. Instead, we utilise the analytic form for the evidence and fit the Dirichlet parameters by maximising this, given a gamma hyperprior distribution for those parameters, this is an example of the ‘evidence framework’ [26]. In practice, this is achieved by coupling an Expectation-Maximisation (EM) algorithm for the Dirichlet mixture parameters with multi-dimensional optimisation of each component's parameters. To answer the crucial question of model fit, we use a Laplace approximation to integrate out the hyperparameters, and estimate the evidence of the complete model. In contrast, the extension to a classifier is relatively simple. We simply fit the model to the different classes, estimate priors as the frequencies of the classes in the training data, and then use Bayes' theorem to calculate the probability that a sample to be classified was generated from each of the classes. We now explain in more detail the model framework and illustrate its utility by application to two example data sets of human gut microbiota [27], [28].

## Materials and Methods

### Multinomial sampling

Our starting point is a matrix of occupancies 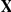 with elements 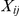 that give the observed abundance of taxa 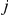 in community sample 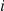 where 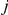 runs from 1 to the total number of taxa 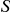, and 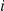 from 1 to the total number of communities 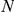. We will denote the rows of this matrix that give the occupancies in each individual community sample by the 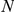 vectors 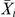. We assume that each community sample is generated from a multinomial distribution with parameter vector 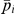. The elements of 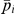, 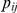, are the probabilities that an individual read taken from community 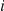 belongs to species 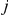. The multinomial distribution corresponds to sampling with replacement from the community. This gives a likelihood for observing each community sample:
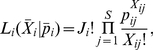
(1)where the 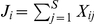 are the total number of reads from each community 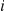. The total likelihood is the product of the community sample likelihoods:
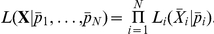



### Dirichlet mixture priors

In a Bayesian approach we now need to define a prior distribution for the multinomial parameter probability vectors 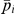. We will refer to these as ‘communities’ since they reflect the underlying structure of the community 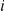 that is sampled. A prior based on the Dirichlet distribution is natural, as it is conjugate to the multinomial and (as we will discuss) has a number of convenient properties. The Dirichlet is a probability distribution over distributions:
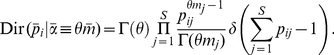
(2)This distribution has 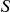 parameters which we can represent as a vector 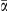 that is a measure i.e. all elements are strictly positive, 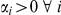. We can express 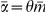, where 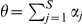 and 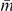 is a normalised measure with 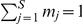. The elements 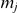 then give the mean 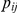 values and the value 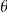 acts like a precision, determining how close the values lie to that mean: a large 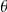 gives little variance about the mean values, while a small 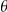 leads to widely distributed samples. Conceptually we view these parameters as describing a ‘metacommunity’, from which different communities can be sampled. The Dirac delta function ensures normalisation, i.e. 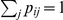.

To provide a more flexible modelling framework and to allow clustering we extend this single Dirichlet prior to a mixture of 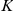 Dirichlets, indexed 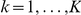, each with parameters 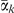 and weight 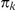 [22], [23]. Each community vector 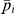 is assumed to derive from a single metacommunity. For each sample 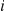, we represent this using a 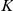-dimensional indicator vector 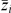 that consists of zeros except for the entry corresponding to the metacommunity that sample 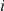 derives from which is equal to one. The prior probabilities for the vectors 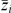 are then just the mixture weights, so:
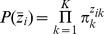
(3)and the complete mixture prior is:
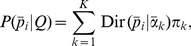
(4)where the Dirichlet distribution is given by Equation 2 , and the mixture prior hyperparameters are 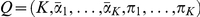.

The numerical behaviour of the model can be improved by placing independent and identically distributed Gamma hyperpriors on the Dirichlet parameters 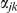, i.e., 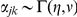. Thus,
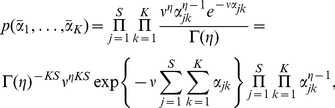
(5)as we will later use the following reparameterisation: 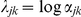, the change of variables formula for probability density functions was used to convert the prior for 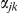 into one for 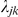, which yields the result that:
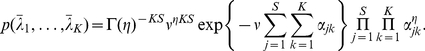
(6)


### Posterior distribution of the multinomial parameters

The posterior distribution of the community parameters is obtained by multiplying the Dirichlet mixture prior by the multinomial likelihood ( Equation 1 ) and appropriately normalising to give for community 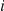:
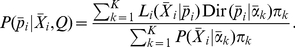
(7)The Dirichlet is a conjugate prior for the multinomial: for a single Dirichlet the posterior is itself a Dirichlet with parameters obtained by summing the observed counts and the Dirichlet parameters, 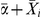. For the Dirichlet mixture this conjugacy is maintained and Equation 7 can also be written as a Dirichlet mixture:
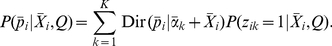
(8)We will discuss the calculation of the posterior probabilities, 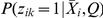, for a sample deriving from a metacommunity below.

### Marginalising the multinomial parameters

The denominator of Equation 7 is equivalent to 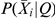, the evidence for community sample 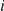. This is obtained by integrating the numerator, i.e. the mixture prior 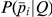 multiplied by the likelihood 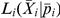, over all possible community priors. It is the complete probability of observing this data marginalising out the unseen vector of probabilities 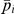. One of the useful properties of the Dirichlet prior is that this evidence has a closed form. So focussing on just a single mixture component 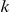:
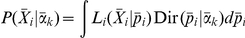


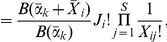
where the function 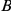 is the multinomial Beta function and can be expressed in terms of Gamma functions as:
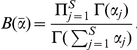
So far we have considered the posterior and evidence for just a single community sample 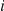. The evidence over all samples is just the product of the evidences for each sample:
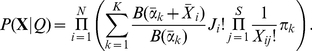
(9)


### EM algorithm for fitting the mixture of Dirichlets prior

Our strategy for fitting the mixture of Dirichlets is to maximise the evidence given the gamma hyperpriors. The strictly Bayesian approach would be to sample from the unobserved hyperparameters, 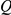, and latent variables 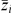, given the hyperpriors, using Markov chain Monte Carlo (MCMC), and then marginalise. This would be computationally challenging for the high dimensional 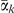 vectors that are encountered in microbiomics data. Maximising the evidence allows us to obtain a single parameter vector that will correspond to the most likely set of parameters given the gamma hyperpriors. The technique is well established and is known as the ‘evidence framework’ [21], [26]. The posterior distribution of the hyperparameters is given by the product of the evidence (Equation 9) and the hyperprior for the 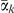 given by Equation 5. Strictly, to distinguish this from the posterior of the multinomial parameters we should refer to this as the marginal posterior distribution but our meaning should be clear from the context used. We are also implicitly assuming uniform hyperpriors for the other components of 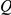, the mixing coefficients 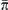. Maximising the posterior of the hyperparameters is equivalent to maximising the log posterior of the hyperparameters, 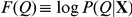. Thus:
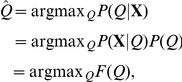
where
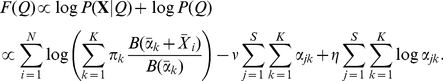
(10)


We now use a binary latent variable matrix 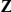 with elements 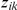 that are 1 if the 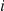
th community sample belongs to the 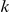
th metacommunity and 0 otherwise. The rows of this matrix are the 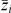 vectors introduced above. This allows us to maximise the log posterior distribution using the popular expectation-maximisation (EM) algorithm [21]. Augmenting the data with these latent variables, the evidence and log posterior distribution, respectively, become:
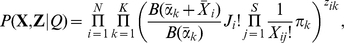


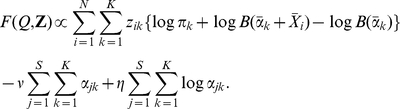
Using Jensen's inequality we obtain a lower bound for the expected log posterior distribution:
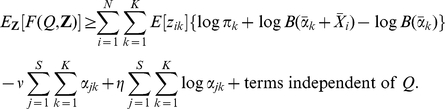
(11)We can calculate 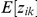 as follows:
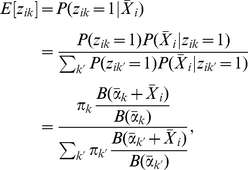
(12)where we have used Bayes' theorem and 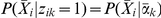.

Following Sjölander *et al* (1996) [22], we now reparameterise and optimise the expected log posterior distribution with respect to these new parameters: to keep the 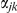
's positive, we set 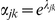, and to keep the 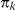
's normalised, we set 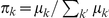. Optimising 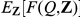 with respect to 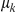 is equivalent to solving the following equation:
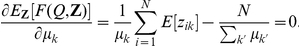
Rearranging this equation we obtain:
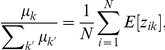
and thus:
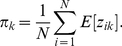
(13)


Our EM algorithm to find 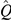 thus alternates between updating the responsibilities 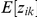, the mixing coefficients 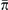 and the Dirichlet parameters 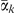, 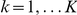:

- Calculate 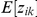 using Equation 12.
- Update 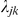 by finding parameters that minimise the negative of Equation 11. In practice we used the Broyden-Fletcher-Goldfarb-Shanno (BFGS) algorithm as implemented in the Gnu Science Library [29].
- Calculate 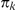 using Equation 13.
- Repeat until convergence of 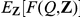, which can be calculated from Equation 11.

We will refer to the hyperparameter values obtained by this method as the maximum posterior estimates (MPE).

#### Model comparison through Laplace approximation

We need to determine the number of components 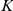 in the Dirichlet mixture. We cannot simply choose the one with the largest log posterior, 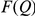, as this takes no account of model complexity: as the number of components is increased, 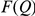 must increase. We could use a heuristic like the Aikaike Information Criterion (AIC) or Bayesian Information Criterion (BIC) to penalise the model parameters but these can give misleading results [21]. Better is to take a fully Bayesian approach to model comparison where probabilities are used to represent uncertainty in the choice of model. Applying Bayes' theorem, the posterior probability of the 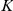 component model 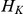 given the data matrix 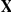 is:
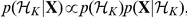
where 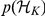 is the prior probability for the 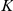 component model, which allows us to express a preference for different models, and 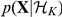 is the model evidence, which expresses the preference of the data for different models. In our case, the model evidence is given by:
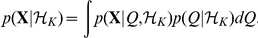
This integral cannot be calculated analytically, but it can be estimated using the Laplace approximation:
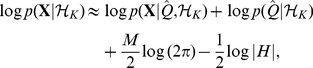
(14)where 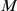 is the number of parameters in 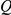, 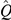 are the parameters maximising the posterior distribution, and 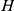 is the Hessian matrix of second derivatives of the negative log posterior evaluated at 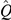:
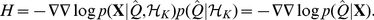
(15)Thus,
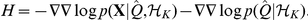



The nonzero elements of the Hessian matrix are given below:
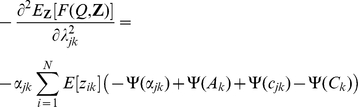


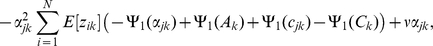


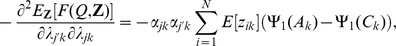
and
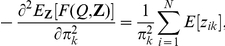
where 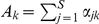, 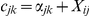, 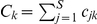, 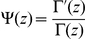 and 
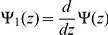
. In the results we will give the negative of Equation 14 so that a better fit corresponds to a smaller value. The Hessian also allows us to calculate uncertainties in the parameter estimates of 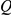, through computing the inverse, then the diagonal elements give the variance of the corresponding parameter.

### Data Sets

#### Twins

To illustrate the application of these ideas to a real data set we reanalysed a study of the gut microbiomes of twins and their mothers [27]. These comprised faecal samples from 154 different individuals characterised by family and body mass index – ‘Lean’, ‘Obese’ and ‘Overweight’. Each individual was sampled at two time points approximately two months apart. The V2 hypervariable region of the 16S rRNA gene was amplified by PCR and then sequenced using 454. We reanalysed this data set filtering the reads, denoising and removing chimeras using the AmpliconNoise pipeline [10], [11]. Denoised reads were then classified to the genus level using the RDP stand-alone classifier [5]. This gave a total of 570,851 reads split over 278 samples since of the 308 possible some failed to possess any reads following filtering. The size of individual samples varied from just 53 to 10,585 with a median of 1,599. A total of 129 different genera were observed with a genera diversity per sample that varied from just 12 to 50 with a median of 28. One extra category ‘Unknown’ was used for those reads that failed to be classified with greater than 50% bootstrap certainty. We will refer to this as the ‘Twins’ data set.

#### IBD

We also include a brief analysis of microbiome data from a study of inflammatory bowel diseases (IBDs) [28]. This comprised faecal samples from 78 individuals where the V5-6 region of the 16S rRNA gene was pyrosequenced using 454. 35 samples were from healthy individuals, 12 from individuals with colonic Crohn's disease (CCD), 15 from individuals exhibiting ileal Crohn's disease (ICD), and 16 from individuals with ulcerative colitis (UC). We processed the data as above. This gave a total of 134,276 reads with individual samples varying in size from 394 to 3,258 with a median of 1,710 reads. 93 separate genera were observed in these samples with a genera diversity per sample that varied from 8 to 33 with a median of 22.

## Results

### Clustering Twins data at the metacommunity level

The mixture of Dirichlets prior can be used to cluster samples at the metacommunity level. Assuming each sample represents a unique community, we can try to infer which metacommunity that community is most likely to have originated from. This is the component for which the posterior probability of each membership is the highest, i.e. the value of 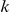 that maximizes 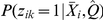 for a particular sample 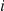. We will denote this value as 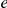. These posterior probabilities will be the equilibrium values of the 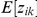 calculated by the EM fitting algorithm.

**Figure 1:**
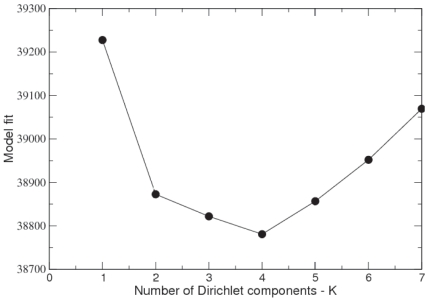
Model fit for mixture of Dirichlets prior to Twins dataset. Evaluates model fit for increasing number of Dirichlet mixture components 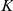 using the Laplace approximation to the negative log model evidence.

To use the mixture of Dirichlets prior for clustering at the metacommunity level we first need to determine what the number of clusters or mixture components 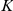 should be. To do this we fitted Dirichlet mixtures by minimising the negative log posterior as described above. To calculate model fit accounting for complexity we then used the Laplace approximation to the model evidence. We did this for increasing values of 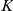 starting with just a single component 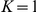. The results are shown in Figure 1 where we see a minimum for 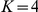 suggesting, firstly, that a mixture of Dirichlets is more appropriate than a single Dirichlet prior for this data set and that, secondly, the mixture has four components.

**Figure 2:**
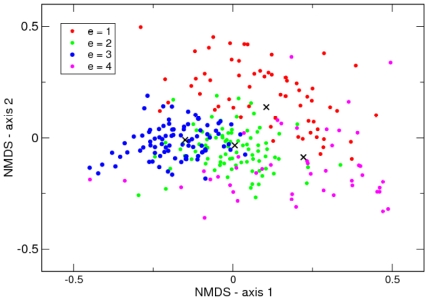
NMDS plot of Twins dataset with hierarchical cluster labellings. Samples arising from each of the four components are shown in red, green, blue and magenta, respectively. The black crosses indicate the Dirichlet means of each component.

The four components have weights 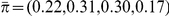. They differ also in how variable their communities are with 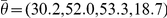. Therefore we have two less abundant highly variable clusters 1 and 4 and two more abundant homogeneous clusters 2 and 3. To graphically illustrate this optimal clustering in Figure 2 we used non-metric multidimensional scaling (NMDS) to generate two-dimensional positions for each community sample, and the mean vectors associated with the four Dirichlet components 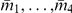, that reflect their Bray-Curtis distances using the isoMDS function of R [30]. From this the higher variability in the first and fourth clusters is readily apparent. Another striking observation is that communities are not necessarily associated with the closest cluster mean. Partially this may reflect imperfect mapping to the two-dimensional space but it will also likely reflect properly accounting for sampling through the multinomial-Dirichlet structure.

To explore the component composition we use the Dirichlet parameter vector obtained by fitting a single mixture to the data set as a reference, which we will denote 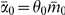. For interest 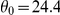 a value that is intermediate to that of the four components. We can get a sense of how different the components are by calculating the sum of their posterior mean absolute differences to the reference 
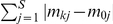
. A quantity which will vary between 0 and 200% for metacommunities that are identical and completely dissimilar to the reference respectively. Calculating this gives 34%, 26%, 51% and 47% for the four components, and a total of 158%, indicating substantial differences in community structures for each component from the reference. How the different OTUs contribute to these differences is shown in Table 1. Comparing the means of the posterior distributions for the four components we find that 30 out of 131 genera account for over 90% of this difference. The Bacteroides alone account for 29% of this difference. This genera is substantially over represented in the third cluster comprising nearly 39% of the community, close to the reference at 23% in the second cluster and observed at much lower proportions in the first and fourth clusters at around 7% and 8%, respectively. The next most significantly different category is actually ‘Unknown’ with nearly 15% more sequences failing to be classified with sufficient confidence in the fourth component, and 8% less in the third component than the reference. Faecilibacterium are substantially under-represented in the fourth component whereas Prevotella is mostly found in the first. The other genera exhibit various patterns but frequently we see over representation in one of or both the first and fourth clusters and little representation in the second and third e.g. Colinsella, Eubacterium, Streptococcus, et cetera.

**Table 1:** Genera frequencies in the Twins Clusters.

Rank	Genus	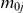	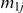	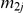	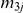	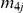	Diff.	C. Diff.
1	Bacteroides	17.5	5-6.8-8	21-22.6-25	35-38.8-43	7-8.3-11	46.3	29.2
2	Unknown	30.8	26-29.1-33	31-33.6-37	20-22.4-25	39-45.2-53	27.2	46.4
3	Faecalibacter.	10.0	12-13.8-16	8-8.8-10	12-13.8-16	3-4.0-5	14.9	55.8
4	Prevotella	0.60	4.2-5.18-6.3	0.2-0.22-0.3	0.1-0.14-0.2	0.2-0.40-0.7	5.6	59.4
5	Alistipes	2.33	1.5-1.86-2.4	3.5-4.02-4.7	1.4-1.66-2.0	0.7-0.99-1.4	4.2	62.0
6	Dorea	2.71	2.7-3.32-4.1	1.2-1.49-1.8	1.4-1.73-2.1	3.1-4.05-5.3	4.1	64.6
7	Ruminococcus	2.05	1.9-2.36-3.0	3.1-3.57-4.2	0.8-0.95-1.2	0.6-0.92-1.4	4.1	67.2
8	Oscillibacter	2.56	2.3-2.84-3.5	3.4-3.96-4.6	1.3-1.59-1.9	0.9-1.20-1.7	4.0	69.7
9	Roseburia	4.13	3.0-3.63-4.5	2.0-2.32-2.8	3.9-4.47-5.2	4.2-5.40-6.9	3.9	72.2
10	Subdoligran.	2.84	2.8-3.40-4.2	2.6-3.04-3.6	1.6-1.91-2.3	1.2-1.62-2.3	2.9	74.0
11	Collinsella	1.37	1.8-2.32-2.9	0.5-0.66-0.8	0.5-0.67-0.9	1.3-1.76-2.5	2.7	75.8
12	Eubacterium	1.03	1.9-2.47-3.1	0.3-0.40-0.5	0.4-0.52-0.7	0.8-1.16-1.6	2.7	77.5
13	Hespellia	1.04	0.4-0.54-0.8	0.5-0.65-0.8	0.5-0.69-0.9	1.4-1.95-2.6	2.1	78.8
14	Coprococcus	2.37	2.3-2.84-3.5	1.6-1.90-2.3	1.1-1.32-1.6	1.7-2.31-3.1	2.1	80.1
15	Streptococcus	1.12	0.9-1.21-1.6	0.4-0.57-0.7	0.5-0.62-0.8	1.2-1.65-2.2	1.7	81.2
16	Coprobacillus	1.13	0.6-0.77-1.1	0.8-0.95-1.2	0.5-0.59-0.8	1.1-1.58-2.2	1.5	82.2
17	Catenibacterium	0.35	0.8-1.09-1.5	0.1-0.09-0.2	0.1-0.15-0.2	0.2-0.30-0.6	1.2	82.9
18	Eggerthella	0.47	0.1-0.24-0.4	0.2-0.30-0.4	0.2-0.22-0.3	0.7-1.00-1.4	1.2	83.7
19	Clostridium	0.74	0.5-0.68-0.9	0.3-0.42-0.6	0.3-0.39-0.5	0.7-1.03-1.5	1.0	84.3
20	Anaerotruncus	1.02	0.8-1.07-1.4	0.7-0.85-1.1	0.4-0.52-0.7	0.5-0.76-1.1	1.0	84.9
21	Odoribacter	0.67	0.6-0.77-1.0	0.5-0.62-0.8	0.2-0.32-0.4	0.1-0.21-0.4	1.0	85.6
22	Barnesiella	0.56	0.5-0.71-1.0	0.5-0.60-0.8	0.2-0.22-0.3	0.1-0.13-0.3	1.0	86.2
23	Megasphaera	0.38	0.5-0.68-1.0	0.1-0.11-0.2	0.1-0.20-0.3	0.3-0.54-0.9	0.9	86.7
24	Paraprevotella	0.29	0.5-0.71-1.0	0.1-0.11-0.2	0.1-0.10-0.2	0.1-0.18-0.4	0.9	87.3
25	Lactobacillus	0.29	0.4-0.60-0.9	0.1-0.12-0.2	0.0-0.08-0.1	0.2-0.40-0.7	0.8	87.8
26	Butyricimonas	0.42	0.4-0.58-0.8	0.2-0.30-0.4	0.1-0.20-0.3	0.1-0.13-0.3	0.8	88.3
27	Butyricicoccus	0.87	0.6-0.79-1.1	0.4-0.47-0.6	0.5-0.60-0.8	0.6-0.84-1.2	0.8	88.8
28	Lactonifactor	0.63	0.5-0.65-0.9	0.3-0.35-0.5	0.3-0.34-0.5	0.6-0.81-1.2	0.8	89.3
29	Parabacteroides	0.77	0.4-0.59-0.8	0.5-0.64-0.8	0.3-0.40-0.5	0.5-0.68-1.0	0.8	89.8
30	Dialister	0.57	0.3-0.49-0.7	0.2-0.27-0.4	0.3-0.42-0.6	0.5-0.78-1.2	0.7	90.2

Percentage relative abundance of the first 30 out of 131 genera in the estimate of the mean of the reference single Dirichlet component, 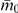, and the four Dirichlet mixture components, 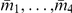 fitted to the Twins data. For the mixture components the upper and lower 95% credible intervals are also given in the format (lower-MPE-upper). These are calculated as the maximum posterior estimate minus/plus two standard deviations as calculated from the inverse Hessian. Genera are ranked in order of their contribution to the total mean difference of 158%, split 34%, 26%, 51% and 47% across components, and the cumulative fraction of this difference accounted for given in the last column in the table.

**Figure 3:**
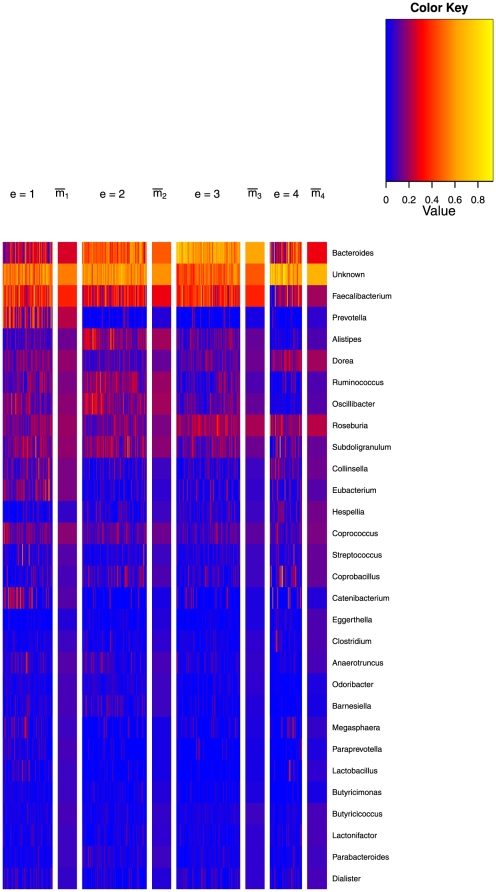
Heat map of the Twins data and hierarchical clustering. Heat map showing the Twins data with samples grouped according to the cluster most likely to have generated them. Only 30 out of 131 genera are shown, those with the greatest variability across clusters, see Table 1. To the right of each cluster the mean of the Dirichlet component for that mixture is shown. The data is square root transformed and therefore to convert the scale to relative abundance, values must be squared.

These patterns are also illustrated graphically in the ‘heat map’ of relative frequencies shown in Figure 3. The relative frequencies of the 30 genera accounting for the most difference between clusters are shown for all the samples. The samples are grouped into the cluster that they had the highest probability of being generated from, as defined above. The cluster means are plotted to the right of the samples mapped to that cluster. Roughly we have that the two low variance clusters are dominated by Bacteroides and Faecilibacterium, albeit to a greater extent in the third cluster. The high variance, first and fourth clusters, contain a greater variety of genera but with substantially more Prevotella and Faecilibacterium in the first, rather than the fourth, where no genus really dominates.

### Generative classifier for Twins data

The Dirichlet-multinomial framework can also be used for classification. This is a supervised learning approach as opposed to the unsupervised approach used in the previous section. Here, we will consider the case of binary classes but any number of classes is a simple extension. Given a training data set of 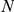 samples 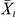 then we denote class membership with the 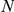 dimensional vector 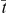 with elements 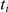 which are either 0 or 1. The classification problem is to deduce the class 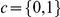 of a new sample 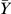. To do this we associate a separate Dirichlet multinomial mixture model with each class. We denote the hyperparameters of these mixtures by 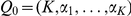 and 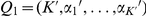, respectively. Then we can marginalise over the multinomial parameters of the sample to be classified so that:
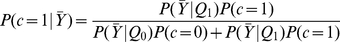
(16)is the probability of the sample belonging to the second class and 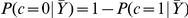. The prior class probabilities are estimated as the observed class frequencies so that 
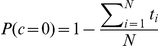
 and 
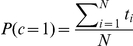
. The class mixture themselves are determined just as before but with data points restricted to those class members. We can also determine if the fit is significant by comparing the sum of model fits of the classes with the model fit ignoring the class variables. This is our generative classification scheme.

**Figure 4:**
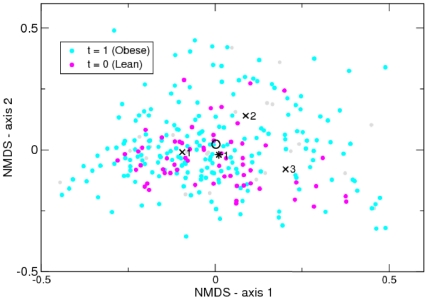
NMDS plot of Twins dataset with class labels. Samples from Lean (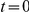) individuals are shown in magenta and Obese (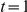) in Cyan. Overweight are grey. The black crosses indicate the Dirichlet means of each component of the three components for the Obese class, the black asterisk the single component for the Lean class. We also show the posterior mean of the entire Obese class as a black circle.

We will apply this to the Twins data denoting individuals with ‘Lean’ BMI by 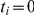 and ‘Obese’ as 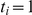. We will ignore the ‘Overweight’ category to avoid ambiguity. In Figure 4 we replot the NMDS plot of Figure 2 with these class labels. There is no dramatic separation of points according to class labels. We found that for the Lean 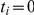 class a single component Dirichlet mixture was optimal but that for the Obese 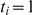 class three components minimised the Laplace approximation to the model evidence. The means of each of the three Obese components were quite different but the posterior mean for the entire prior sampling from all three according to their weights (black circle in Figure 4) is close to the single component from the Lean class (black asterisk Figure 4). In fact, accounting for uncertainty in both the Dirichlet priors and the sampling from those, then only one low frequency genera, Megasphaera, was significantly differently expressed between classes, having a 97% probability of being more abundant in Obese people. In addition, fitting to the two classes separately did not give a significantly better fit than fitting to the whole data set, 35640 vs. 35385. It is also apparent from comparing Figure 2 and Figure 4 that each of the class components map onto one of the components from the clustering of the whole data set, this was confirmed by comparing the Bray-Curtis distances between the two sets of mean vectors, the component from the Lean class maps onto the second of the four from the whole data set, and the three components from the Obese class map onto the third, first and fourth, respectively. In summary, it appears that the difference between Lean and Obese classes lies not at the level of mean community composition but that the Obese individuals contain a greater variety of community structures including three out of the four components found in the complete data set.

In a recent evaluation of classification algorithms applied to microbial community data the random forests algorithm was found to perform best [20], substantially outperforming elastic nets, support vector machines, and multinomial naive Bayes (MNB). The random forests algorithm is an example of ensemble learning where many classifiers are generated and their predictions are aggregated. In particular, it is an extension of the machine learning technique known as bootstrap aggregating or bagging for short. The bagging approach constructs decision trees from bootstrap samples of the data and makes class predictions via majority vote. Random forests adds an extra layer of randomness to bagging by changing how the decision trees are constructed. Instead of splitting each node using the best split amongst all the variables, the best split amongst a subset of randomly chosen predictors is used. Moreover, the random forests algorithm also gives a measure of the importance of a variable by calculating how much prediction error increases when data for that variable is permuted. Random forests therefore seemed like an appropriate benchmark to compare the performance of our generative classifier to. Following Knights et al. (2011) [20], we implemented the random forests algorithm using the randomForest package in R, though we tuned the parameters of the algorithm (the number of variables in the random subset at each node and the number of trees in the forest) according to the heuristics suggested by Liaw and Wiener (2002) [31].

**Figure 5:**
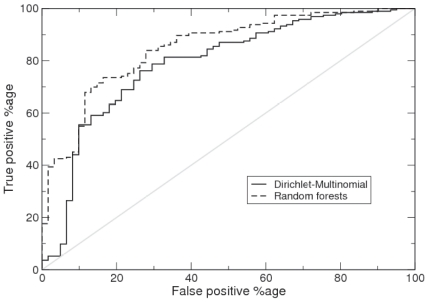
Receiver operating characteristic (ROC) curves for the Twins Dirichlet multinomial and random forests classifiers. Gives true positive percentage on the y-axis i.e. Obese individuals correctly identified vs false positive percentage i.e Lean individuals flagged as Obese.

To compare the two classification methods we performed leave-one-out validation. We removed each sample in turn from the data set, trained the classifier, and classified the missing data point. Assigning the data point as Obese if the predicted probability was greater than or equal to 0.5. We obtained a slightly lower error rate, i.e. fraction of samples misclassified, for the random forests algorithm (18.5%) as opposed to the Dirichlet multinomial generative classifier (22.4%). Examining the ‘confusion matrix’ for each classifier, Table 2, that is the number of individuals from each true class classified into the two classes, reveals that the generative classifier does have a better distribution of errors across classes. We then generated receiver-operating characteristic (ROC) curves for each classifier. These are shown in Figure 5. They are generated by ordering samples by decreasing likelihood of being Obese: for the generative classifier that is simply the probability of being Obese i.e. 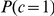; for random forests this is the weighted vote. We then lower a threshold from 1.0 to 0.0 with intervals defined by the sample probabilities. All samples with probability greater than or equal to a given threshold are classified as Obese, all other samples as Lean. Based on these classifications, the false positive percentage (i.e. Lean classified as Obese) and true positive rate (Obese classified as Obese) are calculated and plotted against each other. This is repeated for all thresholds. It is a means of summarising the performance of a classifier over all decision thresholds. Both classifiers do substantially better than random but at lower thresholds random forests outperforms the generative classifier with fewer false positives. A summary statistic is the area under the ROC curve, for random forests this was 85%; for the Dirichlet-Multinomial 79% was obtained.

**Table 2:** Confusion matrices for classification of Twins data.

Predicted	Random forests		Dirichlet multinomial	
Actual	Lean	Obese	Lean	Obese
Lean	19	42	33	28
Obese	5	188	29	164

The two rows give the number of ‘Lean’ and ‘Obese’ individuals predicted to be ‘Lean’ and ‘Obese’ by the random forests and Dirichlet multinomial classifiers following leave-one-out validation. A classification threshold of 0.5 was used for both algorithms.

### Analysis of IBD phenotypes

**Figure 6:**
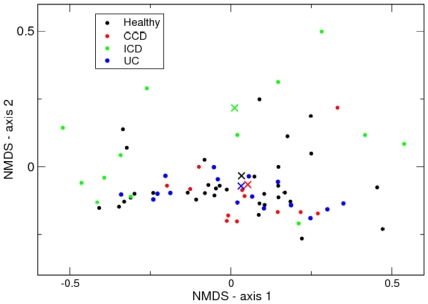
NMDS plot of IBD dataset with class labels. Samples from Healthy individuals (black), and three IBD phenotypes, (red) colonic Crohn's disease (CCD), (green) ileal Crohn's disease (ICD), and (blue) ulcerative colitis (UC) are shown. The Dirichlet means of single component fits to each type are shown by the corresponding coloured cross.

**Figure 7:**
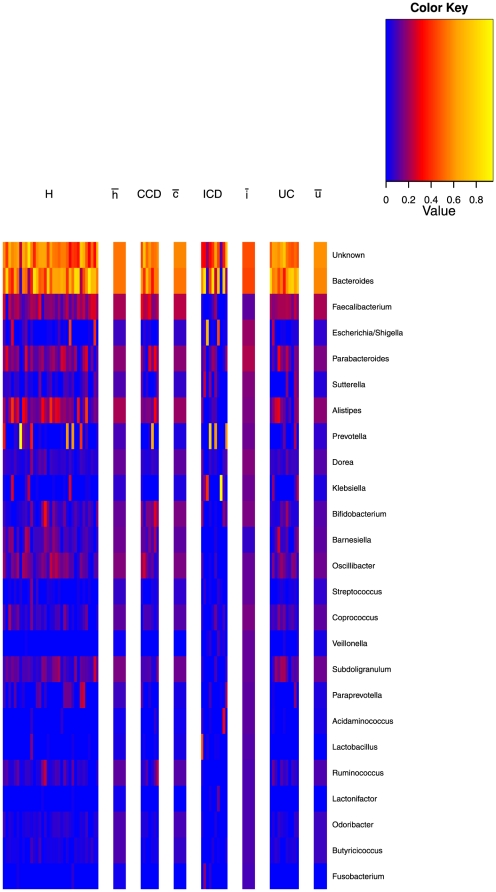
Heat map of the IBD data divided by phenotype together with phenotype means. Heat map showing the IBD data with samples grouped according to the IBD pheonotype. The means of the four single component Dirichlet models, fitted to healthy (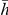), colonic Crohn's disease (CCD - 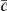), ileal Crohn's disease (ICD - 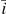), and ulcerative colitis (UC - 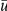) phenotypes are also shown. Only 25 out of 95 genera are shown, those with the greatest variability across phenotypes, see Table 3. The data is square root transformed and therefore to convert the scale to relative abundance, values must be squared.

We conclude with a brief analysis of the inflammatory bowel disease (IBD) phenotypes. In Figure 6 we show an NMDS plot with samples coloured according to phenotype for this data set generated as described above. It is apparent from this that the Healthy (H) individuals, and those exhibiting colonic Crohn's disease (CCD) and ulcerative colitis (UC), have similar, fairly homogeneous community structures whereas the individuals with ileal Crohn's disease (ICD) have a much larger variation in community structure. We can use the DMM model to quantify this, we fitted single component models, to all the samples together, and then each phenotype separately. The 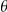 values obtained were 15.7 for the whole data set and (H) 22.2, (CCD) 39.4, (ICD) 5.1, (UC) 38.5 for the phenotypes. Remembering, that 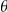 is related to the inverse of the variance, then this confirms that the ICD phenotype is associated with an increase in metacommunity variability. We also show the metacommunity means in Figure 6 as crosses: H, CCD and UC have a similar location whereas the ICD mean is displaced. Exactly how the different OTUs contribute to the differences in the ICD samples is shown in Table 3 and graphically in Figure 7. The proportion of the Unknown, Bacteroides, and Faecalibacterium genera are reduced whereas numerous other genera for example the Escherichia/Shigella, Sutterella, and Prevotella are increased.

**Table 3:** Genera frequencies in the IBD phenotypes.

Rank	Genus	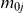	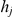	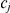	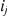	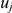	Diff.	C. Diff.
1	Unknown	27.8	24-28.4-34	26-33.4-44	14-21.1-33	27-34.7-44	19.8	18.9
2	Bacteroides	27.2	24-28.7-35	22-28.7-38	12-19.6-31	26-32.5-41	15.9	34.1
3	Faecalibacter.	3.61	3.3-4.36-5.8	3.6-5.39-8.0	0.7-1.52-3.4	3.1-4.46-6.4	5.5	39.4
4	Escherichia/Shigella	0.93	0.3-0.58-1.0	0.2-0.50-1.1	2.1-3.85-7.0	0.2-0.41-0.8	4.2	43.4
5	Parabacteroides	3.49	2.3-3.19-4.3	1.9-3.07-4.9	2.6-4.77-8.6	1.5-2.31-3.6	3.2	46.5
6	Sutterella	1.00	0.6-0.95-1.5	0.2-0.49-1.1	1.3-2.63-5.3	0.1-0.30-0.7	2.9	49.2
7	Alistipes	3.61	3.3-4.41-5.9	2.4-3.79-6.0	1.3-2.56-5.1	1.9-2.92-4.4	2.7	51.8
8	Prevotella	0.77	0.5-0.79-1.3	0.0-0.15-0.6	0.9-1.98-4.4	0.2-0.36-0.8	2.3	54.0
9	Dorea	1.98	1.2-1.65-2.3	0.9-1.48-2.5	1.4-2.70-5.2	0.8-1.29-2.1	2.2	56.2
10	Klebsiella	0.47	0.2-0.34-0.6	0.1-0.21-0.7	0.9-1.95-4.4	0.1-0.16-0.5	2.2	58.2
11	Bifidobacterium	1.91	1.1-1.59-2.3	1.5-2.48-4.1	1.2-2.32-4.7	0.7-1.14-1.9	2.1	60.2
12	Barnesiella	1.65	1.3-1.88-2.7	0.8-1.47-2.6	0.1-0.49-1.9	0.8-1.30-2.2	1.9	62.1
13	Oscillibacter	2.49	2.0-2.75-3.8	1.4-2.31-3.8	0.7-1.60-3.6	1.3-1.97-3.1	1.9	63.9
14	Streptococcus	0.67	0.3-0.44-0.8	0.2-0.44-1.0	0.8-1.76-3.7	0.2-0.37-0.8	1.8	65.6
15	Coprococcus	1.73	0.9-1.37-2.0	0.7-1.20-2.1	1.2-2.41-4.7	0.9-1.46-2.4	1.8	67.4
16	Veillonella	0.17	0.0-0.03-0.2	0.0-0.00-0.0	0.7-1.59-3.6	0.0-0.09-0.4	1.8	69.1
17	Subdoligranulum	2.33	1.8-2.47-3.4	1.2-1.93-3.2	0.7-1.62-3.6	1.2-1.86-3.0	1.7	70.7
18	Paraprevotella	0.55	0.4-0.60-1.0	0.0-0.14-0.5	0.6-1.36-3.3	0.1-0.21-0.6	1.6	72.3
19	Acidaminococcus	0.19	0.0-0.06-0.3	0.0-0.07-0.5	0.6-1.35-3.3	0.0-0.09-0.4	1.5	73.7
20	Lactobacillus	0.20	0.0-0.13-0.3	0.0-0.13-0.5	0.5-1.27-3.1	0.0-0.00-0.0	1.4	75.1
21	Ruminococcus	1.47	1.0-1.40-2.0	0.7-1.29-2.3	0.4-1.01-2.7	0.5-0.94-1.6	1.2	76.3
22	Lactonifactor	0.11	0.0-0.06-0.2	0.0-0.00-0.0	0.4-1.01-2.7	0.0-0.00-0.0	1.2	77.4
23	Odoribacter	1.11	0.7-1.04-1.5	0.4-0.73-1.4	0.3-0.93-2.5	0.4-0.69-1.2	1.0	78.4
24	Butyricicoccus	1.01	0.6-0.92-1.4	0.3-0.53-1.1	0.3-0.93-2.5	0.4-0.66-1.1	1.0	79.3
25	Fusobacterium	0.09	0.0-0.06-0.3	0.0-0.00-0.0	0.3-0.80-2.5	0.0-0.00-0.0	0.9	80.2

Percentage relative abundance of the first 25 out of 95 genera in the estimate of the mean of the reference single Dirichlet component, 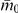, fitted to all IBD individuals, and the four single component Dirichlet models, fitted to healthy (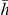), colonic Crohn's disease (CCD - 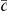), ileal Crohn's disease (ICD - 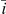), and ulcerative colitis (UC - 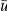) phenotypes. For the mixture components the upper and lower 95% credible intervals are also given in the format (lower-MPE-upper). These are calculated as the maximum log posterior estimate minus/plus two standard deviations as calculated from the inverse Hessian. Genera are ranked in order of their contribution to the total difference of 104% to the reference split 9%, 20%, 48%, 27% across phenotypes, and the cumulative fraction of this difference accounted for given in the last column in the table.

## Discussion

We have demonstrated that the Dirichlet multinomial mixture is a powerful framework for the generative modelling of microbial community data. It operates at several levels, it allows read numbers and hence sampling noise to be naturally accounted for, and the Dirichlet parameters are easily interpretable in terms of the mean and variance of the communities generated from each component. Used for ‘unsupervised learning’ or clustering it provides a means to determine clusters of communities or envirotypes, a highly topical problem in the analysis of microbial community data. Since it is a probabilistic model, we can harness rigorous statistical theory for determining how well the data is explained by a given cluster number.

We illustrated this approach with the Twins data set. Using our models, the most probable estimate for the number of envirotypes present in this sample (or ‘enterotypes’ as they are known in the context of gut microbiota samples) is four. Our measure of model fit, the negative logarithm of the approximate model evidence, was 41 less than the next best cluster number, three. Thus, in the context of our model the probability that there are four rather than three or five clusters is practically a 100%. However, a direct implication of the Bayesian approach is that any point estimate of the number of envirotypes represents a summary (in our case, the mode) of the posterior distribution over the number of clusters. For other data sets the predicted cluster number may be more uncertain. This uncertainty can be naturally incorporated by our approach.

Our analysis, and its statistical implications, may be contrasted with a previous analysis of this same Twins dataset, which used a partitioning around medoid (PAM) clustering coupled with the heuristic Calinski-Harabasz (CH) index [18]. The CH approach makes no acknowledgment of the fact that there is inherent uncertainty in the number of clusters, and thus may potentially be misread as offering an unambiguous and definitive assessment of the number of clusters. Furthermore, the PAM clustering algorithm does not allow clusters to be of variable spread. This may be the reason why they found three rather than four clusters. The extra flexibility of the DMM model could better represent the true patterns in the data. This, to us, supports the promise of a probabilistic model with the flexibility to model clusters of different size and a Bayesian approach to determining the cluster number.

Used for ‘supervised learning’ the Dirichlet multinomial mixture provides an effective classifier. Absolute classification power as summarised by the area under the ROC curve is less than for the best performing of previously tested algorithms - random forests. However, using the standard classification threshold of 0.5 it had a better distribution of errors across classes, outperforming random forests on the smaller ‘Lean’ class. In general, we would expect discriminative classifiers, which only model the conditional probability of the class label given the data, to outperform generative models, which fit the actual class distributions. On the other hand, the generative approach allows much easier interpretation of the fitted models, which is often more important than accuracy *per se*. The fitted Dirichlet parameters describe both the composition of the communities, and critically variance in composition associated with the classes. The probabilistic framework that we present also allows the hypothesis of whether two classes do differ in community composition to be rigorously tested. Or equivalently whether a discrete experimental treatment significantly impacts community structure.

Generative models provide a framework for both clustering and classification but their full power derives from their ability to combine the two. We will illustrate this for the Twins data. In Table 4 we give the proportion of samples from each BMI category, i.e. Lean, Obese and Overweight, that fell into our four enterotypes. For this data set we did not see a significant difference in mean community composition between Lean and Obese individuals. However, it is clear that the two classes do differ significantly in their probability of deriving from each of the clusters. Lean individuals are much less likely to derive from the first and fourth clusters than Obese individuals. They are much more likely to derive from the second and somewhat less likely from the third. This suggests a novel explanation for the differences in taxa frequency that have been previously reported between Lean and Obese individuals from this data. BMI itself is not correlated with changes in community structure rather it influences the likelihood of deriving from the four enterotypes.

**Table 4:** Comparison of BMI and cluster or ‘Enterotype’.

BMI	e = 1	e = 2	e = 3	e = 4
Lean	6.6%	60.7%	24.6%	8.2%
Obese	25.9%	21.2%	33.2%	19.7%
Overweight	29.2%	33.3%	25.0%	12.5%

Proportion of samples with a given BMI deriving from the four enterotypes 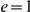, 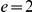, 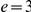, and 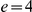.

This raises the intriguing possibility that the first and fourth enterotypes may be associated with a disturbed possibly unhealthy gut microbiota – ‘dysbiosis’. This implies that obesity does not guarantee a disturbed microflora but increases its likelihood. Finally, we return to the observation that the first and fourth enterotypes have a higher variance in community structure than the second and third. We suggest that this is an example of the ‘Anna Karenina principle’ as applied to microbial communities. This principle popularised by Jared Diamond [32] derives from the first line of Tolstoy's novel: “Happy families are all alike; every unhappy family is unhappy in its own way” [33]. We propose that the same thing may apply to microbial communities in human health, there are many more configurations associated with dysbiosis than are possible for a healthy community which is relatively predictable and homogeneous as it requires certain key components. This is not to suggest that the first and fourth enterotypes are associated with higher genera level diversity in individual samples, the median diversities are not significantly different between the enterotypes, it is the diversity in community compositions that increases. Our observations are also consistent, therefore, with the conclusion of the original study that the major impact of obesity was a reduction in OTU diversity [27].

This interpretation of the Twins data is obviously speculative and will require further studies with more meta-data on host health to corroborate. The analysis of the IBD phenotype data represents a first step in this direction. There we did find a much more variable microbiota associated with one of the disease phenotypes, ileal Crohn's disease, but not colonic Crohn's or ulcerative colitis. This is, therefore partial support for the AKP. However, it is possible that the latter two diseases are not strongly associated with gut dysbiosis. Certainly, at the genera level we were unable to discriminate their community compositions from healthy individuals. The number of samples in each of the disease phenotypes was also quite small. We hope that future large-scale sequencing projects will allow us to investigate this question further. The ‘Human Microbiome Project’ is restricted to healthy individuals but that will allow us to verify the existence of the two enterotypes that we propose are associated with a healthy microbiota [34].

The software for fitting the Dirichlet multinomial mixture is available for download from the Google Code project MicrobeDMM (http://code.google.com/p/microbedmm/).
